# Propensity matched analysis of longterm outcomes following transcatheter based aortic valve implantation versus classic aortic valve replacement in patients with previous cardiac surgery

**DOI:** 10.1186/1749-8090-9-99

**Published:** 2014-06-10

**Authors:** Nestoras Papadopoulos, Nina Schiller, Stephan Fichtlscherer, Ralf Lehmann, Christian F Weber, Anton Moritz, Mirko Doss, Andreas Zierer

**Affiliations:** 1 Division of Thoracic and Cardiovascular Surgery, Johann-Wolfgang-Goethe University Frankfurt/Main, Theodor Stern Kai 7, 60590 Frankfurt am Main, Germany; 2 Division of Cardiology, Johann-Wolfgang-Goethe University Frankfurt/Main, Frankfurt am Main, Germany; 3 Clinic of Anesthesiology, Intensive Care Medicine and Pain Therapy, Goethe-University Hospital Frankfurt/Main, Frankfurt am Main, Germany

**Keywords:** Transcatheter based aortic valve implantation, Aortic valve, Aortic valve replacement

## Abstract

**Background:**

The aim of this study was to compare outcome of patients with previous cardiac surgery undergoing transapical aortic valve implantation (Redo-TAVI) to those undergoing classic aortic valve replacement (Redo-AVR) by using propensity analysis.

**Methods:**

From January 2005 through May 2012, 52 high-risk patients underwent Redo-TAVI using a pericardial xenograft fixed within a stainless steel, balloon-expandable stent (Edwards SAPIEN™). During the same period of time 167 patients underwent classic Redo-AVR. Logistic regression analysis was used to identify covariates among 11 baseline patient variables including the type of initial surgery. Using the significant regression coefficients, each patient’s propensity score was calculated, allowing selectively matched subgroups of 40 patients each. Initial surgery included coronary artery bypass grafting in 30 patients, aortic valve replacement in 7 patients and mitral valve reconstruction in 3 patients in each group. Follow-up was 4 ± 2 years and was 100% complete.

**Results:**

Postoperative chest tube drainage (163 ± 214 vs. 562 ± 332 ml/24 h, p = 0.02) and incidence of early permanent neurologic deficit (0 vs. 13%, p = 0.04) was lower in patients with Redo-TAVI and there was a trend towards improved 30-day survival (p = 0.06). Also we detected a decreased ventilation time (p = 0.04) and lower transfusion rate of allogenic blood products (p ≤ 0.05) in the Redo-TAVI group. At late follow up differences regarding incidence of major adverse events, including death and permanent neurologic deficits (25% vs. 43%, p = 0.01) statistically supported early postoperative findings.

**Conclusion:**

The encouraging results regarding early and long-term outcomes following TAVI in patients with previous cardiac surgery show, that this evolving approach may be particularly beneficial in this patient cohort.

## Background

High-risk patients with severe symptomatic degenerative aortic stenosis (AS) who had previously undergone cardiac operation represent a surgically challenging patient cohort [1,2]. Mediastinal reentry during cardiac reoperations especially in patients with previous coronary artery bypass grafting (CABG) bears an elevated risk of myocardial and graft injury and is associated with an increased periprocedural morbidity and mortality [3]. Currently, surgical aortic valve replacement (AVR) represents the gold standard of treatment of degenerative AS with appropriate long-term outcomes [3,4].

Transcatheter based aortic valve implantation (TAVI) has been suggested as an alternative to classic surgery especially in patients with severe comorbidities carrying an unacceptably high perioperative risk and has been demonstrated to be non-inferior to AVR in this patient cohort [5-8]. Due to the minimally invasive nature of this approach that eliminates the need of cardiopulmonary bypass (CPB) and myocardial protection issues, TAVI may be particularly helpful in elderly high risk redo patients.

The aim of this study was to compare outcome of patients with previous cardiac surgery undergoing transapical aortic valve implantation (Redo-TAVI) to those undergoing classic aortic valve replacement (Redo-AVR) by using propensity analysis.

## Methods

### Study design and patient population

This report represents a comparative retrospective single-centre study. Patient data were prospectively collected during treatment using standardized forms to record demographic and clinical characteristics as well as procedural and follow up data. Follow up was obtained postoperatively at 30 days, 6 months and annually based on the medical records and on physician and patient interviews. Since stroke represents a devastating complication following cardiac surgery with a dramatic impairment of quality of life we additionally analyzed the incidence of major adverse events (stroke and death) at late follow-up using Kaplan Meier analysis. All patients underwent transthoracic echocardiography at the time of discharge, at 6 moths, 12 months and yearly thereafter. Full data were obtained in all patients. The mean follow-up was 4 ± 2 years. The local Ethics Committee at the Hospital of the Johann Wolfgang Goethe University, Frankfurt/Main, Germany approved the study protocol and individual patient consent was waived.

Between January 2005 and May 2012, 52 high risk patients underwent Redo-TAVI and 167 Redo-AVR after pervious cardiac surgery. All Redo-TAVI and Redo-AVR procedures were performed at the Division of Thoracic and Cardiovascular Surgery of Johann Wolfgang Goethe University, Frankfurt Main, Germany. Using the significant regression coefficients, each patient’s propensity score was calculated, allowing selectively matched subgroups of 40 patients each. Patient demographics and baseline data of the matched subgroups are reported in Table 1. As a result of our matching process preoperative comorbidities and other important clinical variables were comparable between the two groups. Prior to surgery, the mean transvalvular gradient was considerably increased (Redo-TAVI: 57 ± 21 mmHg vs. Redo-AVR: 51 ± 16 mmHg; p = 0.56) and the aortic valve orifice area (Redo-TAVI: 0.63 ± 0.29 cm vs. Redo-AVR: 0.68 ± 0.31 cm; p = 0.65) severely reduced in both groups.

**Table 1:** Baseline characteristics of the propensity-matched patients of the Redo-TAVI and Redo-AVR group

	**All patients**			**Propensity-matched patients**		
	**Redo-TAVI (n = 52)**	**Redo-AVR (n = 167)**		**Redo-TAVI (n = 40)**	**Redo-AVR (n = 40)**	
**Variables**	**No. (%)**	**No. (%)**	**p value**	**No. (%)**	**No. (%)**	**p value**
Age (years)	82 ± 5	72 ± 9	0.07	81 ± 4	80 ± 3	>0.99
Log EuroSCORE^||^	25 ± 5	17 ± 2	0.11	24 ± 6	19 ± 6	0.58
STS risk score	11 ± 4	9 ± 2	0.07	11.1 ± 2.8	10.4 ± 3	0.65
Male	31 (60)	92 (55)	0.44	29 (73)	29 (73)	>0.99
Arterial hypertension	22 (42)	106 (63)	0.14	16 (40)	18 (45)	0.69
Pulmonary hypertension	16 (31)	72 (43)	0.24	5 (13)	5 (13)	>0.99
Diabetes	20 (38)	43 (26)	0.42	17 (42)	14 (35)	0.54
CAD^‡^	45 (87)	42 (25)	0.03	33 (83)	30 (75)	0.46
COPD^§^	19 (36)	37 (22)	0.67	9 (23)	8 (20)	0.81
Chronic renal failure^*^	30 (58)	21 (12)	0.05	20 (50)	16 (40)	0.43
Peripheral vascular disease	23 (44)	24 (14)	0.04	13 (33)	11 (27)	0.43
Cerebrovascular disease	20 (38)	31 (18)	0.07	9 (23)	8 (20)	0.81
LV-EF < 30^¶^	21 (40)	39 (23)	0.06	9 (23)	9 (23)	>0.99
Mean ejection fraction	39 ± 18	44 ± 14	0.62	48 ± 14	47 ± 12	0.77
Aortic valve area (cm)	0.65 ± 0.24	0.5 ± 0.31	0.46	0.63 ± 0.29	0.68 ± 0.31	0.65
Mean pressure gradient (echo; mmHg)	59 ± 18	57 ± 14	0.59	57 ± 21	51 ± 16	0.56
Peak to peak gradient (invasive; mmHg)	67 ± 27	70 ± 35	0.55	69 ± 36	65 ± 34	0.58
Mean time interval between initialand current cardiac procedure (years)	7 ± 4	11 ± 8	0.60	7 ± 5	8 ± 6	0.78

^‡^CAD = coronary artery disease; ^§^COPD = chronic obstructive pulmonary disease; ^||^EuroSCORE = European system for cardiac risk evaluation; ^¶^LV-EF = left ventricular ejection fraction.

### Patient selection for TAVI in patients with previous cardiac surgery (Redo-TAVI): inclusion criteria

High-risk patients who had previously undergone cardiac operation with severe symptomatic aortic stenosis and an aortic valve orifice area of 0.8 cm^2^ or less were selected for the purpose of this study. The baseline operative risk was estimated by the logistic EuroSCORE and the according STS risk score [9-13]. High risk was defined by a logistic EuroSCORE predicted risk for mortality greater than 20% or a STS risk score greater than 10%. Additional inclusion criteria were an age of 75 years or older and symmetrically distributed calcification of the stenotic native aortic valve cusps. The therapeutic option of Redo-TAVI was discussed extensively with all patients considered suitable for inclusion in the study, focusing on the overall risk profile of the individual patient, on the preoperative activities of daily living, the motivation of the individual patient, and on the ongoing results of the new technique. The choice of treatment was made at the discretion of the heart team, consisting of cardiac surgeons and interventional cardiologists. Preinterventional patient screening included transthoracic and transesophageal echocardiography as well as coronary artery angiography for exclusion of coronary heart disease. Applying these guidelines, we treated 52 consecutive patients who had previously undergone cardiac surgery between January 2005 and May 2012 with an average logistic EuroSCORE predicted risk for mortality of 25 ± 5% and an according STS risk score of 11 ± 4%. Patient demographics of the propensity-matched patients in the Redo-TAVI group are summarized in Table 1.

### Patient selection for Redo-TAVI: exclusion criteria

The presence of one or more of the following comorbidities was considered a contraindication for Redo-TAVI. Echocardiographically evidenced bicuspid aortic valve, noncalcified aortic stenosis, intracardiac thrombus, endocarditis, ejection fraction less than 20%, hypertrophic obstructive cardiomyopathy, untreated symptomatic coronary artery disease, myocardial infarction within less than 1 month and recent stroke.

### Patient population redo surgical aortic valve replacement (Redo-AVR)

During the same period of time (January 2005 - May 2012) 167 consecutive patients with severe aortic stenosis and previous cardiac surgery underwent Redo-AVR. Of these 167 patients the mean logistic EuroSCORE was 17 ± 2% and the according STS risk score was 9 ± 2%. Demographics of propensity-matched patients in the Redo-AVR group are summarized in Table 1.

### Propensity score analysis

The nonrandomness of procedure assignment was addressed by propensity matching to provide a more reliable assessment of outcomes based on procedure type. Logistic regression analysis was used to identify covariates among 11 baseline patient variables that were imbalanced in the 2 groups of interest (SPSS 11.0 for Windows; SPSS Inc, Chicago, Ill). Variables included age, sex, logistic European System for Cardiac Operative Risk Evaluation (EuroSCORE), STS risk score, preoperative left ventricular ejection fraction (LV-EF), chronic obstructive pulmonary disease (COPD), pulmonary hypertension, peripheral vascular disease, cerebrovascular disease, renal dysfunction, type of previous cardiac surgery procedure. By using the significant regression coefficients, a propensity score was calculated for each patient. The total population was ranked by propensity score, allowing selectively matched subgroups of 40 patients each. The short- and long-term outcomes of the patients were blinded during the matching process. Resulting matched patients were analyzed for differences in selected early and late outcomes.

### Procedure: Redo-TAVI

Our institutional protocol for transapical TAVI has been previously described in details [14-16]. Briefly, all operations were performed in a specially equipped angiography suite that fulfils the standards of a hybrid operating room. A monoplane fluoroscopic angiography system (Axiom Sensis; Siemens, Munich, Germany) was used. Besides standard hemodynamic monitoring, transesophageal echocardiography and CPB were routinely available. A limited left anterolateral incision (5–7 cm), in the fifth intercostal space, was used to access the apex of the heart. A bipolar epicardial pacing wire was placed and tested. Two U stitches with Teflon felt pledgets using 3–0 Prolene polypropylene (Ethicon, Inc, Somerville, NJ) were placed in the apex of the left ventricle. They served as a purse string for linear closure of the left ventricle at the end of the procedure. Following balloon valvuloplasty all valve deployments were performed with standard volumetric inflation of the balloon. Fluoroscopy and transesophageal echo were used to guide the catheter across the native valve and direct deployment of the stent at the level of the annulus. During deployment, the heart was unloaded with rapid ventricular pacing. Valve function was immediately assessed by angiographic and echocardiographic visualization. Intercostal blockade was performed with ropivacaine. The pericardium was partially closed over the apex and a left lateral chest tube inserted. The incision was closed in a standard fashion.

### Procedure: Redo-AVR

Our routine institutional protocol for patients with previous cardiac surgery includes dissection of the right axillary artery and placement of a guide wire in the right common femoral vein, prior to repeat sternotomy. Mediastinal structures were dissected and bypass grafts if present identified. After systemic heparinization the axillary artery was cannulated in the majority of Redo-AVR patients (n = 36/40, 90%). Venous cannulation was performed through the right atrial appendage. Cardiopulmonary bypass was initiated at moderate systemic hypothermia (32°C). Cardiac arrest was achieved by instillation of cardioplegic solution into the aortic root and/or retrograde through a cardioplegic catheter placed in the coronary sinus. If present, the patent left internal thoracic artery bypass graft was dissected and occluded during cross clamp time. A left ventricular vent was placed most commonly through the superior right pulmonary vein.

The stenotic native aortic valve or degenerated aortic prosthesis was excised and aortic valve replaced in a standard fashion. A biologic prosthesis was used in all patients with a mean size of 24 ± 2 mm (range, 19 to 27 mm).

### Data analysis

Data are presented as frequency distributions and percentages. All continuous data are expressed as means ± standard deviation. Categoric data are expressed as counts and proportions. Comparisons were done with paired, 2-tailed *t* test for means of normally distributed continuous variables and the Wilcoxon rank sum test for skewed data. Fisher exact or χ^2^ test was used to analyze differences among categoric data. For long-term survival, Kaplan-Meier estimates were calculated and compared using the log-rang test. Statistical analysis of data was conducted with the SPSS system for statistics (SPSS 11.0 for Windows, SPSS Inc).

## Results

### Surgical data

In both groups, all cases were performed as elective procedures. Duration of procedure was significantly lower in the Redo-TAVI group (106 ± 53 vs. 332.5 ± 120 min; p = 0.01). One patient (3%) in the Redo-TAVI group required temporary CPB support because of hemodynamic instability after completion of Redo-TAVI procedure. Two further patients (5%) in the Redo-TAVI group received percutaneous coronary angioplasty. Table 2 summarizes procedural characteristics of both groups.

**Table 2:** Procedural characteristics of the propensity-matched patients of the Redo-TAVI and Redo-AVR group

		**Propensity-matched patients**				
		**Redo-TAVI (n = 40)**		**Redo-AVR (n = 40)**		**p value**
		**No.**	**%**	**No.**	**%**	
Priority of procedure							
Elective		40	100	40	100	>0.99	
Duration of surgery/intervention (min)		106 ± 53		332.5 ± 120		0.01	
CPB time^*^					171 ± 77		
Cross clamp time				98 ± 42			
Device Redo-TAVI							
Edwards SAPIEN 23 mm		26	65				
Edwards SAPIEN 26 mm		14	35				
Valve type Redo-AVR							
Carpentier Edwards Porcine Valve							
	19 mm			1	3		
	21 mm			7	17		
	23 mm			9	23		
	25 mm			19	47		
	27 mm			4	10		
Immediate result							
Successful valve/device implantation		40	100	40	100	>0.99	
CBP support		1	3	40	100	>0.001	
Additional procedures							
PCI^†^		2	5	0	0	0.18	

^*^CPB = Cardiopulmonary Bypass; ^†^PCI = Percutaneous coronary intervention; TAVI = Transapical aortic valve implantation.

### Post-operative course and early outcomes

**Figure 1:**
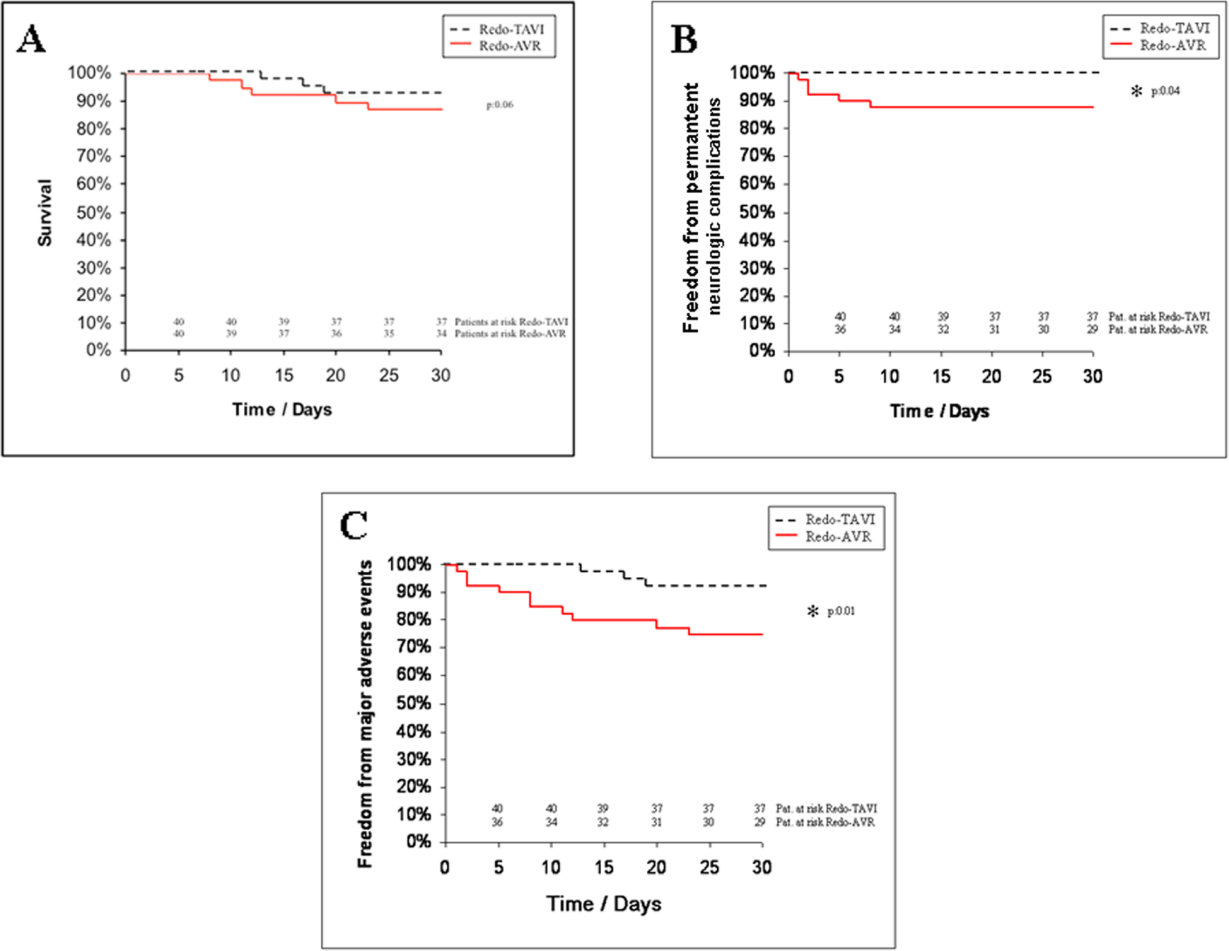
**Time to event curves for early survival (A), early freedom from permanent neurologic complications (B) and early freedom from adverse events (C).** Events were calculated with the use of Kaplan Meier methods. Redo-TAVI denotes transcatheter based aortic valve implantation after previous cardiac surgery and Redo-AVR surgical aortic valve replacement after previous cardiac surgery.

The clinical course of both groups is detailed in Table 3. Postoperative chest tube drainage was significantly lower in the Redo-TAVI group at 24 hours (mean 163 ± 214 vs. mean 562 ± 332 ml/24 h; p = 0.02). We detected a decreased need of transfusion rate of allogenic blood products, including packed red blood cell concentrates (1 ± 1 U vs. 6 ± 6 U; p = 0.04), fresh-frozen plasma (1 ± 1 U vs. 4 ± 3 U; p = 0.05) and platelet concentrates (0 U vs. 4 ± 1 U; p = 0.01) in the Redo-TAVI group. Accordingly the need of reexploration for bleeding was decreased in the Redo-TAVI group (3% vs. 17%; p = 0.05). In patients with Redo-TAVI, ventilation time (p = 0.04) and intensive care unit stay (p = 0.03) were lower as compared to the Redo-AVR group. Implantation of a permanent pacemaker due to atrioventricular block was not necessary in the Redo TAVI group. In contrast 3 patients required permanent pacemaker implantation following Redo-AVR (8%; p = 0.31).Permanent neurologic deficits including stroke and intracranial bleeding were detected with an incidence of 13% (n = 5) in the Redo-AVR group while no such major neurologic events occurred in the Redo-TAVI group (p = 0.04). There was a trend towards a decreased 30 day mortality following Redo-TAVI (p = 0.06). The incidence of major adverse events including death and permanent neurologic complications within 30 days postoperatively reached statistical significance between the two groups and is depicted in Figure 1.

**Table 3:** The detailed clinical postoperative course of the propensity-matched patients of the Redo-TAVI and Redo-AVR group

	**All patients**					**Propensity-matched patients**				
	**Redo-TAVI (n = 52)**		**Redo-AVR (n = 167)**		**p value**	**Redo-TAVI (n = 40)**		**Redo-AVR (n = 40)**		**p value**
**Variables**	**No.**	**(%)**	**No.**	**(%)**		**No.**	**(%)**	**No.**	**(%)**	
Ventilation time (h)	8 ± 6		18 ± 24		0.07	9 ± 7		31 ± 41		0.04
ICU stay (h)^†^	25 ± 21		65 ± 68		0.04	23 ± 20		74 ± 97		0.03
Postoperative complications										
Bleeding complications										
Postop. chest tube drainage (ml/24 h)^§^	144 ± 209		580 ± 420		0.05	163 ± 214		562 ± 332		0.02
Surgical reexploration	2 (4)		13 (8)		0.09	1 (3)		7 (17)		0.05
Transfusion rate of allogenic										
Blood products										
PRBC (U)^||^	1 ± 1		6 ± 6		0.03	1 ± 1		6 ± 6		0.04
FFP (U)^*^	1 ± 1		5 ± 5		0.04	1 ± 1		4 ± 3		0.05
PC (U)^‡^	0		4 ± 2		0.01	0		4 ± 1		0.01
Low cardiac output	2 (4)		4 (2)		0.23	2 (5)		2 (5)		>0.99
Acute kidney injury										
Creatinin increase >300%	3 (5)		6 (4)		0.18	1 (3)		2 (5)		0.09
Permanent PM implantation^^^	1 (2)		5 (3)		0.21	0		3 (8)		0.31
Cerebrovascular										
TIA^¶^	2 (4)		4 (2)		0.23	1 (3)		2 (5)		0.09
Major stroke (Rankin score >2)	0		7 (4)		0.07	0		5 (13)		0.04
Wound healing disorder										
Conservative treatment	0		11 (7)		0.04	0		2 (5)		0.06
Surgical treatment	0		6 (4)		0.05	0		1 (3)		0.07
Thirty day mortality	3 (6)		14 (8)		0.14	3 (8)		6 (16)		0.06

^*^FFP = fresh-frozen plasma; ^†^ICU = intensive care unit; ^‡^PC = Platelet cells; ^§^Postop. = Postoperative; ^||^PRBC = packed red blood cells; ^^^PM = pace maker; ^¶^TIA = Transient ischemic attack.

### Late follow up

**Figure 2:**
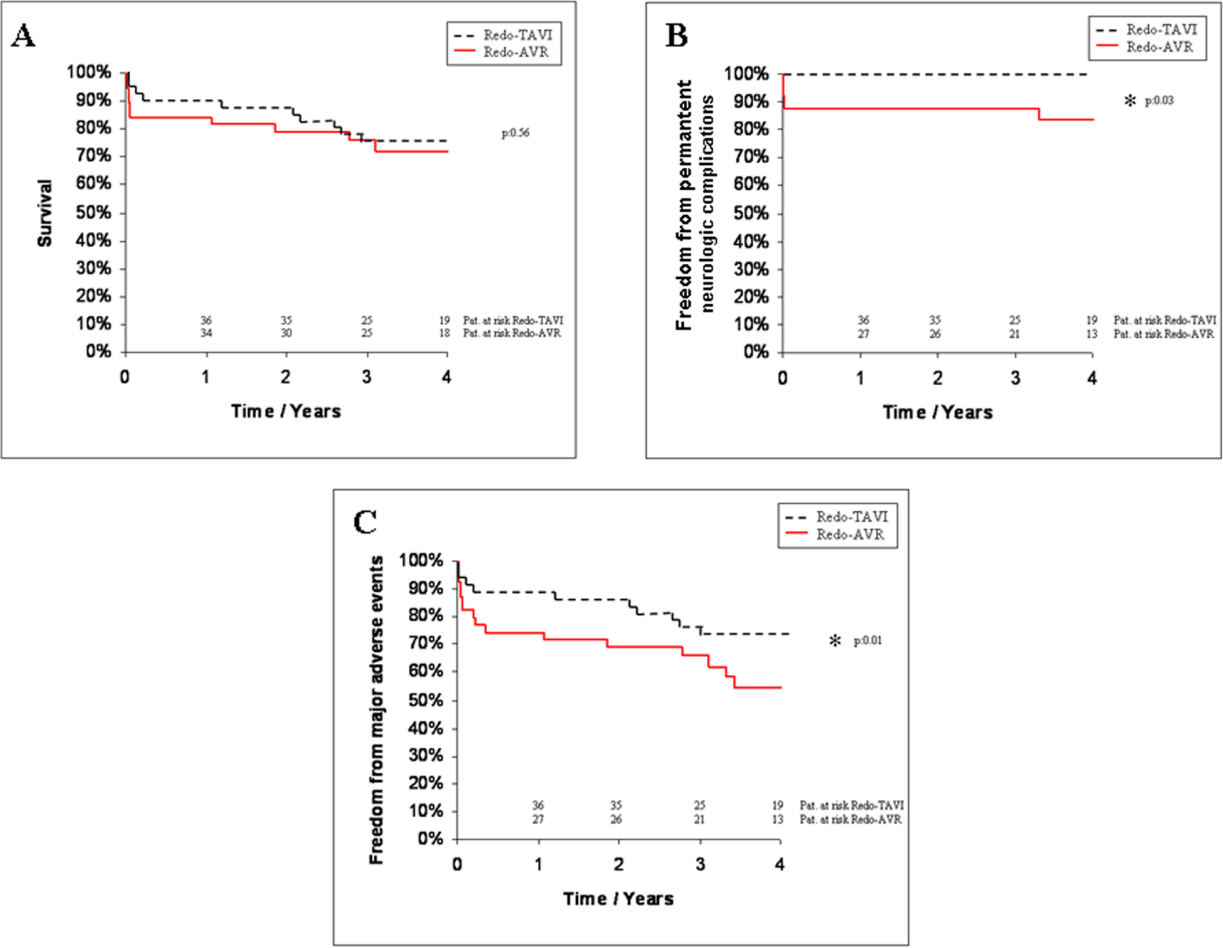
**Time to event curves for late survival (A), late freedom from permanent neurologic events (B) and freedom from adverse events (C).** Events were calculated with the use of Kaplan Meier methods. Redo-TAVI denotes transcatheter based aortic valve implantation after previous cardiac surgery and Redo-AVR surgical aortic valve replacement after previous cardiac surgery.

During late follow up 7 patients in the Redo-TAVI group and 5 patients (p > 0.99) in the Redo-AVR group died. The causes of late death in the transapical group were sepsis (n: 3), cancer (n: 3) and unknown (n: 1). In the surgical group 2 patients died for unknown reasons 13 and 31 months postoperatively. Two further patients of Redo-AVR group died due to pneumonia and consequent respiratory failure and one patient died due to pulmonary malignoma. Actuarial survival rates for all patients are illustrated in Figure 2. Kaplan Meier survival estimates at 4 years were 73 ± 4% for Redo-AVR patients and 75 ± 3% (p = 0.56) for the Redo-TAVI group.While 1 patient (3%) from Redo-AVR suffered a permanent neurologic complication during the follow-up time, late stroke was absent in the Redo-TAVI group (p > 0.99). Overall none of the patients in the Redo-TAVI group suffered a stroke up to 4 years after valve implantation (Figure 2). Kaplan Meier analysis of major adverse events showed a significantly lower incidence at 4 year follow up for the transcatheter group (n: 10, 25%) compared to the surgical group (n: 17, 43%; p = 0.01).

A summary of echocardiographic results at late follow up can be found in Table 4. Briefly, late echocardiographic follow-up data revealed good valve function with low transvalvular gradients in both groups. Ventricular function was stable in both groups. Major difference between the two groups was the presence of mild to moderate paravalvular leakage in 33% of Redo-TAVI patients, while this finding was absent in the Redo-AVR group (p = 0.001). Interestingly, none of the 7 Redo-TAVI patients who underwent a valve in a valve procedure revealed paravalvular leakage in echocardiographic follow-up. During follow-up there were no cases of endocarditis or structural valve degeneration in either group.

**Table 4:** Echocardiographic data at late follow-up in patients underwent Redo-TAVI and Redo-AVR

	**Propensity-matched patients**				
	**Redo-TAVI (n = 40)**		**Redo-AVR (n = 40)**		**p value**
	**No. %**		**No. %**		
Left ventricular ejection fraction	56 ± 4		58 ± 2		0.65
Mean transvalvular gradient (mmHg)	9.8 ± 3		10.1 ± 2		0.71
Effective orifice area (cm^2^)	1.5 ± 0.7		1.4 ± 0.5		0.78
Paravalvular leaks					
Grade I/II	13	33	0	0	0.001
Grade III/IV	0	0	0	0	>0.99

## Discussion

There is no doubt that cardiac surgery is facing the challenge of increasing patient age and comorbidities, both of which lead to a higher perioperative risk profile [17]. Transcatheter based aortic valve implantation is evolving rapidly and provides a reliable and attractive alternative to the established gold standard of classic AVR in patients with severe comorbidities carrying an unacceptably high perioperative risk [5]. The aim of the current study was to compare outcome of patients with previous cardiac surgery undergoing transapical aortic valve implantation (Redo-TAVI) to those undergoing classic aortic valve replacement (Redo-AVR) by using propensity analysis.

The analysis of postoperative data regarding mortality revealed differences inherent in the two treatment options. Regarding early operative outcomes, there was a trend towards a higher 30 day mortality following Redo-AVR as compared to Redo-TAVI patients (p = 0.06). Challenging technical issues during redo surgery increase the surgical trauma which may lead to a complicated postoperative course and reported 30 day mortality following Redo-AVR to be as high as 20% depending on the preoperative risk profile of the investigated patient cohort [5,7,18-20]. Vohra et al. reported a 30 day mortality of 8% in 104 patients undergoing Redo-AVR [1]. Similar results were observed by Dobrilovic and colleagues, with a reported mortality of 6% in a series of 132 patients undergoing Redo-AVR [21]. On the other hand the results of a subgroup analysis in the PARTNER trial revealed an early mortality of 19% for patients who underwent AVR after previous CABG [5]. Although experienced surgeons would find early mortality following Redo-AVR of 16% too high, our observed 30 day mortality within the Redo-AVR group seems to be in line with data reported in previous series [1,5,7,18-21]. For the Redo-TAVI group our results with respect to 30 day mortality can be considered good as compared to the literature. D’Onofrio and colleagues reported in a recent publication about a 30 day mortality of 7% in 110 patients undergoing TAVI after previous cardiac surgery [2]. Walther and colleagues reported a 30 day mortality of 12% in a series of 25 high risk patients undergoing TAVI due to symptomatic aortic valve stenosis after previous cardiac surgical interventions [22].

Besides early mortality permanent neurologic deficit remain a concern in high risk patients with a history of previous cardiac surgery. Transcatheter based aortic valve implantation as analyzed, via the transapical approach, for the purpose of the current study may substantially reduce or even abandon this devastating complication as evidenced in a 0% stroke rate in our series. In the open surgical group a significantly higher rate of permanent postoperative neurologic events were observed (13%; p = 0.04). Stortecky and colleagues assessed in a recent publication the perioperative clinical outcome of patients undergoing Redo-AVR or Redo-TAVI in a retrospective comparative study of two randomly selected, non matched subgroups of 40 patients each [20]. In their series they demonstrated a lower incidence of stroke in the Redo-TAVI group. However this difference didn’t reach statistical significance. The reported incidence of stroke in their series was 3% (n = 1). Such a low rate of permanent neurologic complications is in accordance with previously published data reporting an incidence of stroke of 0 to 1% after Redo-TAVI [2,7]. Similarly to our data Ducroq et al. explored the early and mid-term outcome of 54 patients undergoing TAVI after previous CABG reporting excelent results with an early stroke rate of 0% [7]. The improvement of the incidence of permanent neurologic complications with a perioperative risk of close to 0% certainly represents a powerful advantage of this evolving approach.

In general, surgical trauma is reduced due to the less invasive nature of TAVI as pictured by elimination of the need for mediastinal reentry, extensive adhesiolysis, and the abolishment of extracorporal circulation. Statistically this reduced surgical trauma is reflected in lower postoperative chest tube drainage and a lower transfusion rate of allogenic blood products in the Redo-TAVI group. Similar results were observed by Strotecky and colleagues, with a reported significantly lower requirement of blood components per patients in the Redo-TAVI group compared to Redo-AVR [20].

Due to steadily increasing patient age and life expectancy we learned from previous studies that even in octogenarians not only early operative outcomes but also mid-term and late follow up data are crucial to adequately compare TAVI to classic AVR [16]. During late follow-up, both groups were similar with respect to mortality and new onset of neurologic events. Evaluation of the actuarial survival after a mean follow up of 4 ± 2 years reveals comparable results regarding survival rate between the two groups (75% vs. 73%, p = 0.56). In contrast the incidence of major cerebrovascular events at 4 year follow up was higher in the Redo-AVR group (15% vs. 0%, p = 0.03). As a major finding of our study, we identified the difference regarding the incidence of major adverse events, including death and major neurologic complications (25% vs. 43%, p = 0.01).

Echocardiographic data at 4 years revealed similar results with respect to left ventricular function, aortic valve orifice area, and transvalvular gradients for both groups. Thirteen Redo-TAVI patients (33%) demonstrated mild to moderate paravalvular insufficiency, while no patient in the Redo-AVR group showed any paravalvular regurgitation (p = 0.001). The observed rate of paravalvular leakage is in accordance with previously published data [16,20]. Results recently published from the PARTNER trial investigator group showed that the presence of paravalvular leakage was associated with increased late mortality [6]. In our series we could not find such a correlation, probably due to the small number of patients investigated in the current study [6]. Interestingly, in our series none of the 7 patients (18%) in the Redo-TAVI group, who underwent valve in a valve procedure revealed paravalvular leakage. This may be related to the additional safety net of an existing aortic bioprosthesis, which prevents the risk of annular rupture and allows a more aggressive valve deployment. Another survival benefit of Redo-TAVI patients may be due to the presence of patent CABG-grafts which precludes the risk of coronary obstruction following valve implantation. Nonetheless efforts to reduce the rate of paravalvular leakage following TAVI with specific treatment options or device features in second generation treatment valves are currently being evaluated [23-26].

The encouraging findings in patients undergoing transcatheter based aortic valve implantations in previous series have facilitated the ongoing investigation of patients with a lower risk profile in the PARTNER II trial. We are convinced that future guidelines for the proper patient selection for TAVI should not only consider patient age and comorbidities bad also technical details such as the presence of previous cardiac surgical interventions.

## Conclusions

In conclusion, our data support outcomes reported in the literature following Redo-AVR to treat valvular aortic stenosis in high-risk patients with history of previous cardiac surgery. The encouraging results regarding early and long-term outcomes following TAVI in patients with previous cardiac surgery show, that this evolving approach may be particularly beneficial in this patient cohort.

## Abbreviations

AS: Aortic stenosis; AVR: Aortic valve replacement; CABG: Coronary artery bypass grafting; CPB: Cardiopulmonary bypass; EuroSCORE: European system for cardiac risk evaluation; ICU: Intensive care unit; PCI: Percutaneous coronary intervention; Redo-AVR: Classic aortic valve replacement after previous cardiac surgery; Redo-TAVI: Transcatheter aortic valve implantation after previous cardiac surgery; TAVI: Transcatheter aortic valve implantation.

## Competing interests

M. Doss, S. Fichtlscherer and R. Lehmann are consultants for Edwards. The other authors have no conflicts of interest to declare.

## Authors’ contributions

NP wrote the manuscript and performed together with NS and AZ the statistical analysis. CFW participated in the design of the study. SF, RL, MD, AM and AZ performed the Redo-TAVI and Redo-AVR procedure and conceived tothe study, and participated in its design and coordination and help to draft the manuscript. All authors read and approved the final manuscript.

## References

[B1] Vohra HA Pousios D Whistance RN Haw MP Barlow CW Ohri SK Livesey SA Tsang GM Aortic valve replacement in patients with previous coronary artery bypass grafting: 10-year experience Eur J Cardiothorac Surg 2012 9 1 6 10.1093/ejcts/ezr055 22219478

[B2] D’Onofrio A Rubino P Fusari M Musumeci F Rinaldi M Alfieri O Gerosa G on behalf of the I-TA investigators Impact of previous cardiac operations on patients undergoing transapical aortic valve implantation: results from the Italian Registry of Transapical Aortic Valve Implantation Eur J Cardiothorac Surg 2012 9 480 485 10.1093/ejcts/ezs027 22351707

[B3] Walther T Blumenstein J van Linden A Kempfert J Contemporary management of aortic stenosis: surgical aortic valve replacement remains the gold standard Heart 2012 9 23 29 10.1136/heartjnl-2012-302399 23143122

[B4] Bonow RO Carabello BA Chatterjee K de Leon AC Jr FDP Freed MD Gaasch WH Lytle BW Nishimura RA O’Gara PT O’Rourke RA Otto CM Shah PM Shanewise JS American College of Cardiology/American Heart Association Task Force on Practice Guidelines American College of Cardiology/American Heart Association Task Force on Practice Guidelines. 2008 focused update incorporated into the ACC/AHA 2006 guidelines for the management of patients with valvular heart disease J Am Coll Cardiol 2008 9 1 142 10.1016/j.jacc.2008.03.036 2947945

[B5] Smith CR Leon MB Mack MJ Miller DC Moses JW Svensson LG Tuzcu EM Webb JG Fontana GP Makkar RR Williams M Dewey T Kapadia S Babaliaros V Thourani VH Corso P Pichard AD Bavaria JE Herrmann HC Akin JJ Anderson WN Wang D Pocock SJ PARTNER Trial Investigators Transcatheter versus surgical aortic-valve replacement in high-risk patients N Engl J Med 2011 9 2187 2198 10.1056/NEJMoa1103510 21639811

[B6] Kodali SK Williams MR Smith CR Two-year outcomes after transcatheter or surgical aortic-valve replacement N Engl J Med 2012 9 1686 1695 10.1056/NEJMoa1200384 22443479

[B7] Ducrocq G Al-Attar N Himbert D Messika-Zeitoun D Iung B Descoutures F Nataf P Vahanian A Early and mid-term outcomes in patients undergoing transcatheter aortic valve implantation after previous coronary artery bypass grafting Eur J Cardiothorac Surg 2012 9 499 504 10.1093/ejcts/ezr041 22345175

[B8] Walther T Simon P Dewey T Wimmer-Greinecker G Falk V Kasimir MT Doss M Borger MA Schuler G Glogar D Fehske W Wolner E Mohr FW Mack M Transapical minimally invasive aortic valve implantation: multicenter experience Circulation 2007 9 240 245 10.1161/CIRCULATIONAHA.106.677237 17846311

[B9] Roques F Nashef SA Michel P Gauducheau E de Vincentiis C Baudet E Cortina J David M Faichney A Gabrielle F Gams E Harjula A Jones MT Pintor PP Salamon R Thulin L Risk factors and outcome in European cardiac surgery: analysis of the EuroSCORE multinational database of 19030 patients Eur J Cardiothorac Surg 1999 9 816 822 10.1016/S1010-7940(99)00106-2 10431864

[B10] Shahian DM O’Brien SM Filardo G Ferraris VA Haan CK Rich JB Normand SL DeLong ER Shewan CM Dokholyan RS Peterson ED Edwards FH Anderson RP Society of Thoracic Surgeons Quality Measurement Task Force The Society of Thoracic Surgeons 2008 cardiac surgery risk models: part 1- coronary artery bypass grafting surgery Ann Thorac Surg 2009 9 2 22 10.1016/j.athoracsur.2009.05.053 19559822

[B11] O’Brien SM Shahian DM Filardo G Ferraris VA Haan CK Rich JB Normand SL DeLong ER Shewan CM Dokholyan RS Peterson ED Edwards FH Anderson RP Society of Thoracic Surgeons Quality Measurement Task Force The Society of Thoracic Surgeons 2008 cardiac surgery risk models: part 2- isolated valve surgery Ann Thorac Surg 2009 9 23 42 10.1016/j.athoracsur.2009.03.086 19559823

[B12] Shahian DM O’Brien SM Filardo G Ferraris VA Haan CK Rich JB Normand SL DeLong ER Shewan CM Dokholyan RS Peterson ED Edwards FH Anderson RP Society of Thoracic Surgeons Quality Measurement Task Force The Society of Thoracic Surgeons 2008 cardiac surgery risk models: part 3-valve plus coronary artery bypass grafting surgery Ann Thorac Surg 2009 9 43 62 10.1016/j.athoracsur.2009.05.055 19559824

[B13] Shahian DM He X Jacobs JP Rankin JS Welke KF Filardo G Shewan CM O’Brien SM The Society of Thoracic Surgeons Isolated Aortic Valve Replacement (AVR) Composite Score: a report of the STS Quality Measurement Task Force Ann Thorac Surg 2012 9 2166 2171 10.1016/j.athoracsur.2012.08.120 23127768

[B14] Zierer A Wimmer-Greinecker G Martens S Moritz A Doss M The transapical approach for aortic valve implantation J Thorac Cardiovasc Surg 2008 9 948 953 10.1016/j.jtcvs.2008.06.028 18954635

[B15] Zierer A Wimmer-Greinecker G Martens S Moritz A Doss M Is transapical aortic valve implantation really less invasive than minimally invasive aortic valve replacement? J Thorac Cardiovasc Surg 2009 9 1067 1072 10.1016/j.jtcvs.2009.04.057 19740493

[B16] Doss M Buhr EB Martens S Moritz A Zierer A Transcatheter-based aortic valve implantations at midterm: what happened to our initial patients? Ann Thorac Surg 2012 9 1400 1406 10.1016/j.athoracsur.2012.05.051 22776084

[B17] Gummert JF Funkat A Beckmann A Ernst M Hekmat K Beyersdorf F Schiller W Cardiac surgery in Germany during 2010. A report on behalf of the German Society for Thoracic and Cardiovascular Surgery Thorac Cardiovasc Surg 2011 9 259 267 10.1055/s-0030-1271191 21667446

[B18] Redlich K Khaladj N Peterss S Pichlmaier M Shrestha M Hoy L Haverich A Hagl C Conventional aortic valve replacement in patients with concomitant coronary artery disease and previous coronary artery bypass grafting in the era of interventional approaches Eur J Cardiothorac Surg 2011 9 455 462 21256760 10.1016/j.ejcts.2010.11.067

[B19] Fighali SF Avendaño A Elayda MA Lee VV Hernandez C Siero V Leachman RD Cooley DA Early and late mortality of patients undergoing aortic valve replacement after previous coronary artery bypass graft surgery Circulation 1995 9 163 168 10.1161/01.CIR.92.9.163 7586402

[B20] Stortecky S Brinks H Wenaweser P Huber C Pilgrim T Windecker S Carrel T Kadner A Transcatheter aortic valve implantation or surgical aortic valve replacement as redo procedure after prior coronary artery bypass grafting Ann Thorac Surg 2011 9 1324 1330 10.1016/j.athoracsur.2011.05.106 21880298

[B21] Dobrilovic N Fingleton JG Maslow A Machan J Feng W Casey P Sellke FW Singh AK Midterm outcomes of patients undergoing aortic valve replacement after previous coronary artery bypass grafting Eur J Cardiothorac Surg 2012 9 819 825 10.1093/ejcts/ezs070 22495353

[B22] Walther T Falk V Borger MA Kempfert J Ender J Linke A Schuler G Mohr FW Transapical aortic valve implantation in patients requiring redo surgery Eur J Cardiothorac Surg 2009 9 231 234 10.1016/j.ejcts.2009.02.016 19324566

[B23] Kempfert J Rastan AJ Beyersdorf F Schönburg M Schuler G Sorg S Mohr FW Walther T Trans-apical aortic valve implantation using a new self-expandable bioprosthesis: initial outcomes Eur J Cardiothorac Surg 2011 9 1114 1119 21924620 10.1016/j.ejcts.2011.01.078

[B24] Treede H Mohr FW Baldus S Rastan A Ensminger S Arnold M Kempfert J Figulla HR Transapical transcatheter aortic valve implantation using the JenaValve™ system: acute and 30-day results of the multicentre CE-mark study Eur J Cardiothorac Surg 2012 9 131 138 10.1093/ejcts/ezs129 22508111

[B25] Lerakis S Hayek SS Douglas PS Paravalvular aortic leak after transcatheter aortic valve replacement: current knowledge Circulation 2013 9 397 407 10.1161/CIRCULATIONAHA.112.142000 23339094

[B26] Généreux P Head SJ Hahn R Daneault B Kodali S Williams MR van Mieghem NM Alu MC Serruys PW Kappetein AP Leon MB Paravalvular leak after transcatheter aortic valve replacement: The New Achilles’ Heel? a comprehensive review of the literature J Am Coll Cardiol 2013 9 1125 1136 23375925 10.1016/j.jacc.2012.08.1039

